# The impact and cost-effectiveness of the Amref Health Africa-Smile Train Cleft Lip and Palate Surgical Repair Programme in Eastern and Central Africa

**DOI:** 10.11604/pamj.2017.28.35.10344

**Published:** 2017-09-14

**Authors:** Hasan Hamze, Asrat Mengiste, Jane Carter

**Affiliations:** 1 School of Public Health, University of Alberta, 11405-87 Ave Edmonton, Alberta, Canada; 2 Medical Services Programme, Amref Health Africa Headquarters, PO Box 27691-00506 Nairobi, Kenya

**Keywords:** Burden of disease, cleft lip and palate, reconstructive surgery, cost-effectiveness, economic benefit, cost-benefit

## Abstract

**Introduction:**

Cleft lip with or without cleft palate (CLP) is a congenital malformation that causes significant morbidity in low and middle income countries. Amref Health Africa has partnered with Smile Train to provide CLP surgeries since 2006.

**Methods:**

We analyzed anonymized data of 37,274 CLP patients from the Smile Train database operated on in eastern and central Africa between 2006 and 2014. Cases were analyzed by age, gender, country and surgery type. The impact of cleft surgery was determined by measuring averted Disability-Adjusted Life Years (DALYs) and delayed averted DALYs. We used mean Smile Train costs to calculate cost-effectiveness. We calculated economic benefit using the human capital approach and Value of Statistical Life (VSL) methods.

**Results:**

The median age at time of primary surgery was 5.4 years. A total of 207,879 DALYs were averted at a total estimated cost of US$13 million. Mean averted DALYs per patient were 5.6, and mean cost per averted DALY was $62.8. Total delayed burden of disease from late age at surgery was 36,352 DALYs. Surgical correction resulted in $292 million in economic gain using the human capital approach and $2.4 billion using VSL methods.

**Conclusion:**

Cleft surgery is a cost-effective intervention to reduce disability and increase economic productivity in eastern and central Africa. Dedicated programs that provide essential CLP surgery can produce substantial clinical and economic benefits.

## Introduction

Cleft lip with or without cleft palate (CLP) is one of the most common external congenital anomalies in the world, with an incidence of about 1 in 700 births in eastern Africa [1]. Cleft lip (CL) is a disfiguring condition and cleft palate (CP) causes substantial morbidity related to feeding, communication, and speech development. In terms of social impact, CLP creates concern for parents and social isolation from the family and community. In some parts of the world, such as in Sub-Saharan Africa, infanticide may also occur [2]. Correcting CLP through surgery is relatively straightforward, although specialized surgical expertise and good post-operative facilities are required to avoid potentially lethal complications of surgery. This has created a significant surgical backlog in many low- and middle-income countries (LMICs) where expertise and infrastructure may not be readily available [3]. As a result, large numbers of children with CLP remain untreated for many years in LMICs, some into adulthood.

Historically, the belief that surgery is too resource-exhaustive to be conducted in a sustainable manner has limited the development of surgical subspecialty care in LMICs [4]. Nonetheless, plastic and reconstructive surgeons have taken the lead in addressing surgical disease through organizations such as Interplast, Operation Smile, and Smile Train. These organizations have shown that in light of the broad social and functional disabilities that accompany CLP, these conditions can be repaired for a reasonable financial cost [5]. Additionally, an increasing number of studies are beginning to contradict the long-held view that surgery is too costly to be sustained in LMICs; in fact, recent studies have shown that well-executed surgical programs can provide cost-effective and economically beneficial care even in limited-resource settings [4, 6–8]. Therefore, surgery is gradually being recognized as a vital component and partner of global health efforts to alleviate suffering in LMIC.

Beyond cost, the significant unmet need for CLP surgical correction is also due to lack of surgical expertise in LMICs as well as the lesser priority given to non-emergency surgical conditions such as CLP. There is also a lack of research about the impact and cost-effectiveness of reconstructive surgery outreach programs in eastern and central Africa specifically. Impact and cost-effectiveness are typically expressed through disability-adjusted life year (DALY) values, which are the metrics used by the World Health Organization (WHO) to estimate the burden of disease in the Global Burden of Disease (GBD) study [9].

The DALY is a useful tool with which to compare health outcomes and has become essential for setting priorities, particularly in LMICs. However, DALYs have little meaning when presented as a concept in isolation [5]. When combined with burden of disease measurements, cost-effectiveness analysis generates $/DALY values for specific interventions, allowing for direct comparisons amongst them. The use of$/DALY measures demonstrate that the cost-effectiveness of surgical services in LMICs countries is comparable to that of some non-surgical interventions such as vaccines and oral rehydration programs [10]. Recent work has demonstrated that it can also be economically beneficial in resource-poor settings [4]. Magee et al. [11], Corlew [12] and Moon et al. [13] determined that the cost per DALY averted for CLP repair at the hospital level in developing countries is indeed comparable with other diseases commonly addressed by the global health community. There is currently little evidence of the impact and cost effectiveness of CLP surgery in eastern and central Africa. Therefore, the objective of this study is to estimate the averted and delayed averted burden of cleft disease, cost-effectiveness and economic benefit of the work of Amref Health Africa and SmileTrain in eastern and central Africa using WHO’s GBD methodology.

## Methods

### Data collection

To quantify the possible impact and economic benefit of CLP repair, we used an economic modeling approach based on retrospective data from January 2006 to May 2014. Amref Health Africa, one of the largest Africa-based non-governmental organizations working in medical and health-related development, partners with Smile Train USA to provide CLP reconstructive surgeries to babies, children and adults [14]. Through this program, more than 3^7^,000 cleft disease surgical interventions have been conducted in eight countries (Burundi, Democratic Republic of Congo, Ethiopia, Kenya, Rwanda, South Sudan, Tanzania, Uganda) in eastern and central Africa from January 2006 to May 2014.

The Smile Train Express cleft care database has been used to store collected patient data electronically for surgeries funded by Smile Train. This data represents the largest data set concerning CLP surgery in eastern and central Africa, and documents information on the patient’s cleft condition, general health conditions, and family history. The data set was extracted and anonymized from the current Smile Train Express database of all cleft surgical procedures. It was accessed in Excel format and included age, sex, country, and type of surgery.

### Calculating averted DALYs and delayed averted DALYs

DALYs are calculated as the sum of the years of life lost due to premature mortality (YLL) and years lived with disability (YLD). They are calculated using the disability weights (DW) assigned for untreated and treated cleft deformities by the GBD study. Uncorrected, the DW are 0.09^8^ for CL and 0.231 for CP, which treatment reduces to 0.016 and 0.015, respectively [15]. Since DWs for lip-nose revision (LNR) and palatine fistula (PF) closure have not yet been established, they were estimated to be 0.082, similar to the DW for CL. Therefore, LNR and PF patients were grouped with CL patients under one category for the purposes of calculating clinical and economic impact. For the patients who had both CL and CP, the DW for CP was used. Because the 2004 GBD study does not assign disability weights for secondary cleft disorders, we included only primary cases in the analysis. Standard WHO life expectancies for each age-gender group were assigned from a current life table for each country.

DALYs were calculated both with and without the concepts of discounting and age weighting [15]. To signify whether DALYs have been adjusted for age-weighting and discounting, the symbols DALYs [r,K,β] are used, where r is the discount rate, K is the modulation of age-weighting, and β is the age-weighing parameter. DALYs [0,0,0] signifies no age-weighting and no discounting, whereas DALYs [3,1,0.04] signifies full age-weighting at β=0.04 and 3% discounting rate. Delayed averted burden due to surgical backlog was calculated using the following formula [3]: delayed averted burden = (actual age - ideal age at surgery) x DW. Where actual age pertains to the age at first surgical correction performed for each patient and ideal age pertains to international surgical standards of practice (6 months of age for CL repair and 12 months for CP) [3].

### Cost-effectiveness analysis

Cost-effectiveness analysis (CEA) was performed using mean averted DALYs per procedure and the Smile Train reimbursement rate for cleft surgery. This rate ($350 USD per patient) covers all surgery-related expenses with no cost ascribed to the patient. This includes all fixed costs pertaining to hospital operation and physician salaries. The total cost for all CLP patients was obtained by multiplying the number of CLP cases by the average Smile Train reimbursement rate for cleft surgery. Then, the sum of total costs for the CLP cases was divided by the number of DALYS averted (with and without age-weighting and discounting) to calculate the cost per DALY averted as a result of CLP surgery.

CEA is a usual companion to burden of disease work and has numerous methodologies. The WHO has suggested thresholds for determining whether an intervention is cost-effective based on work by the Commission on Macroeconomics and Health [16]. An intervention that costs less than the GDP/capita per DALY averted is considered very cost-effective. An intervention that costs between one and three times the GDP/capita per DALY is still cost-effective, but an intervention that costs more than three times the GDP/capita per DALY is considered not cost-effective [4, 17]. We used the same parameters in this study to determine whether an intervention is cost-effective.

### Calculating economic benefit

WHO and others have outlined a basic methodology for approximating the potential economic benefit of treating a specific disease, through the translation of DALYs into dollars [5, 12, 17]. Economic benefit of cleft disease repair for each country in eastern Africa was determined using two different methodologies. Using the first method, the country-specific DALYs were multiplied by the corresponding Gross National Income per capita (GNI/capita) for both DALYs calculated without age-weighting and discounting, and DALYs calculated with age-weighting and 3% discounting. The GNI/capita for each country in 2011 were obtained from the World Bank using the Purchasing Power Parity (PPP) method. The PPP method was used instead of the Atlas method for calculating GNI/capita, as it accounts better for differences in comparative price levels across countries and thus is likely to result in a more valid cross-country measure of income per capita [5].

When presenting the results using a human capital methodology (GNI/capita [PPP]), a range of economic benefits is given. As discounting and age-weighting decrease total DALYs attributable to a disease, the lower bound is calculated by multiplying DALYs [3,1,0.04] by GNI/capita (PPP), and the upper bound is calculated by multiplying DALYs [0,0,0] by GNI/capita (PPP).

Economic benefit was also determined using country-specific estimates of Value of Statistical Life (VSL) and age-weights consistent with those estimates. To determine the VSL for each eastern and central African country involved in the outreach, we used the following equation:

$$\text{VSL(CEA) = VSL(USA) }Χ\;{\left[\frac{GNIp.c.(CEA)}{GNIp.c.(USA)}\right]}^{IE-VSL}$$

Where VSL (CEA) is the value of a statistical life in a country in eastern/central Africa, VSL(USA) is the value of a statistical life in the United States ($9.1 million), GNI p.c.(CEA) is the GNI/capita in a country in central or eastern Africa, and GNI p.c. (USA) is the GNI/ capita in United States ($52,340). IE-VSL is income elasticity and as it increases, the estimated VSL in LMICs decreases. IE-VSL values of 0.55-1.0 are most often used in transferring estimates of VSL; however, recent evidence suggests that higher values are more appropriate for conversions to low-income countries [18]. Therefore, VSL is estimated based on IE-VSL values of 1.0 and 1.5.

When presenting the results using a VSL methodology, a range of economic benefits is given. The lower bound is calculated by multiplying IE-VSL = 1.5 by DALYs ~β and the upper bound is calculated by multiplying IE-VSL = 1.0 by DALYs ~β. The benefit-cost ratio (BCR) was calculated by dividing the estimated economic benefit accrued by the estimated total costs of the program.

### Statistical analysis

Simple descriptive statistics (totals and averages) were generated from the demographic variables, DALYs, CEA, and economic benefit results using Microsoft Excel software.

## Results

### Clinical impact and cost-effectiveness

**Figure 1:**
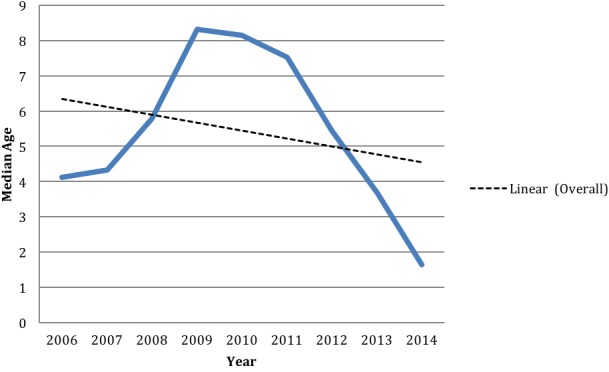
Median age of CLP patients operated on, by year

Patient demographic and clinical information is listed in Table 1. A total of 37,274 patients underwent CLP surgical operations between 2006 and 2014. Thirty-eight percent of the patients were female, and seventy-nine percent of the patients were in the pediatric age range. The median age at primary surgery was 5.38 years, which decreased between 2006 and 2014 as depicted by the linear fit line in Figure 1.

**Table 1:** Patient demographics

Characteristic	Value
No. of Patients	37,274
**Age, yr**	
Median, IQR	5.38, 1.1-14.9
Mean ± SD	10.1 ± 12.1
Number of pediatric patients (≤18 years of age), n(%)	29,938 (78.8)
Number of adult patients (>18 years of age), n(%)	7,336 (21.2)
Female, n (%)	14,189 (38.0)
**Operation type n (%)**	
Combo (cleft lip and cleft palate)	1,037 (2.8)
Isolated cleft lip	29,981 (80.4)
Isolated cleft palate	3,591 (9.6)
Lip nose revision	1848 (5.0)
Palatal fistula	817 (2.2)
**Sex split (Males), %**	
All procedures	62
Isolated cleft palate	61

Averted DALYs, delayed averted DALYs, and cost-effectiveness for CLP surgical correction are shown in Table 2. Surgical correction of CLP resulted in a total of 122,359-207,879 averted DALYs, depending on age-weighting and discounting parameters. The total cost estimated for the procedures performed was US$13,045,900. The sensitivity analysis carried out for averted DALYs per procedure was then used to calculate the cost per DALY, resulting in $62.76-$106.62 for CLP repair. The total delayed averted burden of cleft disease due to advanced age at surgery is shown in Table 2, and was estimated at 31,601-36,352 DALYs over eight years.

**Table 2:** Averted DALYs, delayed averted DALYs and cost-effectiveness ($/DALY averted)

Variable	Unadjusted0,0^+^	Adjusted3,1,0.04^+^
**Cleft lip**		
DALYs averted	150,391	88,732
DALYs averted per patient	4.61	2.72
Cost (USD)	11,426,100	11,426,100
$/DALY averted	75.98	128.77
**Cleft palate**		
DALYs averted	57488	33627
DALYs averted per patient	12.42	7.27
Cost (USD)	1,619,800	1,619,800
$/DALY averted	28.18	48.17
**Total**		
DALYs averted	207,879	122,359
DALYs averted per patient	5.58	3.28
Cost (USD)	13,045,900	13,045,900
$/DALY averted	62.76	106.62
Delayed averted DALYs	36,352	31,601

+ The nomenclature disability-adjusted life-years (r, K, β) is used to signify whether disability-adjusted life-years have been adjusted for discounting or age-weighting, where r is the discount rate, K is the modulation of age-weighting, and β is the age-weighting parameter. Disability-adjusted life-years (3, 1, 0.04) denotes a 3 percent discount rate, with age-weighting at 4 percent.

### Economic impact *human capital methodology*

Using the human capital approach, the total economic benefit of repairing CLP from 2006–2014 was $171.0 million–$291.8 million. For CL and CP, the potential benefit of intervention ranged from $121.1 million to $206.1 million and $49.9 million to $85.7 million, respectively. This resulted in an economic gain of $4,588-$7,828 per CLP patient (Table 3).

**Table 3:** Economic gain for all CLP surgeries using GNI/Capita and VSL Methods

Variable	Human capital		VSL (3,1, ~β)^+^	
	Unadjusted (0,0^+^)	Adjusted (3,1,0.04)^+^	IE-VSL=1.0	IE-VSL=1.5
**Cleft lip**				
Economic gain (USD)	$206,050,770	$121,059,210	$1,682,830,500	$279,048,472
Economic gain per patient	$6,311.67	$3,708.24	$51,547.83	$8,547.71
Cleft Palate				
Economic gain (USD)	$85,738,170	$49,936,770	$700,794,405	$120,960,879
Economic gain per patient	$18,525.97	$10,790.14	$151,424.89	$26,136.75
**Total**				
Economic gain (USD)	$291,788,940	$170,995,980	$2,383,624,904	$400,009,352
Economic gain per patient	$7,828.22	$4,587.54	$63,948.73	$10,731.59

+ VSL, value of a statistical life; IE-VSL, income elasticity of value of a statistical life.

+ The nomenclature disability-adjusted life-years (*r, K, β*) is used to signify whether disability-adjusted life-years have been adjusted for discounting or age-weighting, where *r* is the discount rate, *K* is the modulation of age-weighting, and *β* is the age-weighting parameter. Disability-adjusted life-years (3, 1, 0.04) denotes a 3 percent discount rate, with age-weighting at 4 percent. ~β denotes country specific age-weighting parameter based on life expectancy.

### Value of a statistical life methodology

Using the VSL approach, the total economic benefit of repairing CLP from 2006–2014 is $400.0 million-$2.4 billion, resulting in an economic gain of $10,732-$63,949 per CLP patient (Table 3). Considering an estimated total cost of US$13,045,900 and between $400.0 million-$2.4 billion economic gain using the VSL approach, the estimated BCR ranges from 30.1-184.

## Discussion

This study provides evidence for the clinical and economic impact of surgery for cleft lip and palate in eastern and central Africa. Based on previous estimates in LMICs [4, 5, 19], we hypothesized that a surgical program for cleft repair can yield substantial clinical and economic benefits in this region.

Indeed, Amref Health Africa and Smile Train’s cleft lip and palate surgical program have produced a substantial clinical benefit. Surgeons performed a total of 37,274 CLP surgeries over eight years in the eastern and central African regions.

The sex split for patients undergoing CLP procedures favors males (62%), which is consistent with the literature (60–67%) [20]. However, the sex split for patients is higher than expected for isolated CP (62%), where the expected male ratio is only 33% [3, 20–22]. This may suggest that sex bias may affect families’ decisions to seek care for children with less visible deformities such as CP [3].

The average age at CLP surgery may be used as a surrogate marker of surgical backlog. Surgical backlog can be defined as the numbers of patients in a population who were operated on at an age greater than the standard for that procedure [3]. The mean age for all Smile Train patients operated on in eastern and central Africa was 10.1 years. This was predictable, as a previous study has demonstrated that African patients were the oldest at surgery (mean 9.8 years) compared to patients in other continents [3].

Between 2006 and 2010, the median age of patients undergoing CLP surgery increased from 4.1 to 8.1 years (Figure 1). This is likely because early in each program, procedures were performed at central health facilities, where the mean age of patients was already low [3, 23]. As the program expanded to remote areas, the mean age increased due to the large numbers of older patients with CLP. From 2011 to mid-2014, the median age steadily decreased from 7.5 to 1.6 years, suggesting the alleviation of surgical backlog for CLP in the eastern and central African region.

Our results demonstrate a lower rate of CP repair compared to CL in eastern and central Africa. Indeed, it has been previously reported that Africa has a significantly lower rate of CP repair [3, 24–27]. In comparison, the balance between CL and CP procedures in Asia, the Americas, and eastern Europe is similar, resembling incidence statistics for the condition [3, 27]. This disparity has several likely explanations: first, CP, unlike isolated CL, can be fatal in infants. This “hidden mortality” results in fewer CP operations [28]. Patients are also more likely to access care for visible, disfiguring conditions such as CL than for hidden, functional deformities such as CP. Also, surgery for CP is more challenging than for CL, requiring more advanced surgical expertise and better postoperative care [3]. As Smile Train disbursement rates are the same for CL repair and CP repair, the incentive to repair CP may be diminished [3, 29].

In terms of impact, our study focused on the ‘met need’ (averted DALYs) and ‘unmet need’ (delayed averted DALYs). The calculations confirmed the large burden of disease averted through Smile Train funding in eastern and central Africa. Whereas the DALY values correlated closely with numbers of procedures, the mean DALY values per procedure were low, but consistent with findings from other studies in LMICs [3–5, 13]. This was due to advanced age at surgery and lower life expectancy in the region.

Our data also allowed estimation of the delayed averted burden caused by the YLD before surgery. This delayed averted burden is part of the unmet need, and may represent the shortcomings of the program in its inability to deliver surgical interventions in a timely manner [30]. It is well known that timely closure of CP is associated with improved speech outcome [29, 31]. Delay in care on the other hand may be associated with more difficult procedures, higher likelihood of complications, and poor outcomes [3, 29, 32, 33]. Additionally, delayed repair of CLP can lead to impaired family and societal relationships, with potential long-term psychological effects on the child [29, 34, 35].

Overall, CEA shows that CLP operations conducted through Smile Train funding from 2006– 2014 cost $63-$107 per DALY averted. Our $/DALY figures place CLP surgery as very cost-effective according to WHO cost-effectiveness thresholds. For even the poorest countries, the upper cost of $107 represents only a fraction of the gross domestic product (GDP) per capita according to the WHO guidelines. Our data thus support the cost-effectiveness of specialized surgery and greater resource allocation toward pediatric surgical care in LMICs.

Several recent publications have also explored the cost-effectiveness of CLP surgical missions [3, 11–13]. Poenaru measured cost-effectiveness of Smile Train in alleviating the global burden of cleft disease, estimating the mean cost per DALY between $72 and $134 [3]. Magee et al. assessed the cost-effectiveness of eight Operation Smile missions, estimating the costs of primary cleft surgeries at $796 (using standard life tables) or at $34 after extending disability beyond the age of 5 years [11]. Using clinical data for 568 patients treated in 2005 from a permanent Interplast program in Nepal, Corlew estimated the cost per DALY between $29 and $79 [12]. Moon et al. assessed the cost-effectiveness of CLP surgery performed during Smile for Children’s missions in Vietnam, estimating the cost per DALY averted over a 4-year period between $43 and $79, depending on the year of mission [13]. A benefit of $/DALY figures is that they facilitate the direct comparison of the cost-effectiveness of different interventions. Estimates of $64–$107 per DALY compare favorably with other public health interventions in LMICs [9, 12, 36].

Economic modeling to translate DALYs averted from CLP surgery into 2014 U.S. dollars revealed a significant economic impact. CLP surgery resulted in between $171.0 million and $2.4 billion in economic gain, depending on the modeling approach. This translates into between $4,588 (human capital) and $63,949 (VSL) in economic gain per patient. While this appears to be a large number, it is important to recognize that this represents a significant portion of the economic productivity for the majority of patients. As previously mentioned, cleft disease results in cultural rejection in many parts of eastern and central Africa, and those with the disease remain unemployed for the majority of their lifetime.

Any approach to economic modeling carries a set of implicit assumptions. The human capital approach typically represents a conservative estimate of an intervention’s impact primarily because it assigns value based solely on contributions to the economy. It uses GNI per capita as a proxy for personal economic productivity and assumes that the value of improved health varies in relation to the value of that person’s labor [4, 17]. It does not account for personal valuations of risk reduction. By comparison, the VSL approach typically represents the higher end of economic modeling estimates. VSL estimates are based on human behavior and are thus likely to provide more accurate assessments of the personal value assigned to health risk reductions [4, 37]. The VSL approach has been used by governmental agencies around the world in benefit-to-cost analyses and has been applied to evaluate public health interventions at the community level [4, 38]. We believe that the economic impact of investment in surgical programs for CLP in LMICs likely lies nearer to the VSL estimate as it accounts for personal valuations of health risk reduction [4].

Our results compare favorably with other studies that have attempted to estimate the economic gain of CLP treatment in terms of U.S. dollars. Corlew determined that the economic impact of CLP repair yielded between $48.2 and $120.0 million in economic benefit [12]. This translates to between $2,620-$143,363 per CL patient and between $7,013- $375,412 per CP patient. In contrast to this previous study, we constructed our VSL economic model using country-specific data to ensure that both DALYs and VSL values peak at two-thirds of a country’s life expectancy [39]. Such estimates are able to provide a more region-specific and internally consistent estimate of the total economic impact of surgical intervention within that community [4]. Using this method of economic modeling, Hughes et al. estimated that the DALYs averted from CLP surgery into 2011 U.S. dollars resulted in between $4.7 million and $27.5 million, or between $46,456 and $269,227 per patient in economic gain, depending on the modeling approach [4].

This study also derives a BCR for CLP repair in eastern and central Africa. Our findings estimate a BCR of 30 to 184, using the more conservative estimate of economic benefit. These findings suggest that investment in CLP repair is a good economic proposition with a net positive return on investment.

### Limitations

A better understanding of the morbidity associated with CL and CP would be valuable. As previously discussed, morbidities associated with CLP include speech and feeding difficulties. Further research is needed to determine the severity and prevalence of associated morbidities, as well as the ability to alleviate problems associated with CLP as the age of the patient at the time of surgery increases.

DWs for procedures not included in the GBD study, such as for LNR and PF, are the author’s estimates. Further, we chose to assign the CP DW for patients who underwent a combination CL/CP surgery. It is likely that the DW for both conditions combined is higher than the DW of CP alone, therefore we may have underestimated the DALYs averted for such patients. As such, validation of DW values for such conditions would allow a more accurate estimate of DALYs.

Another limitation of our study is that the cost data used in this study did not include additional lifelong costs for speech therapy and other rehabilitation expenses. A better understanding of the cost implications to the hospital hosting the mission as well as the cost to patients and families accessing care would help create a more holistic understanding of the cost of the intervention [40].

The lack of complete costing profile only allows us to estimate gross benefits rather than net benefits. Accurate and reliable cost estimates for cleft centers and short-term programs could provide valuable information for BCA to better determine the scale of investment benefits and to contextualize plastic surgery within other global health initiatives [40].

## Conclusion

Cleft surgeries are cost-effective interventions to reduce the burden of disease in eastern and central Africa. Dedicated programs that provide essential CLP surgery can produce substantial clinical and economic benefits. Our results demonstrate that further investment is justified to reduce the burden of this disability in eastern and central Africa. Our results also demonstrate that well executed surgical programs can be cost-effective and as such should be included in global health discussions on reducing the burden of disease in LMICs. Further study into the BCR of surgical programs in LMICs would allow for meaningful comparisons, as there are currently no publications on this subject.

### What is known about this topic

- There is a substantial burden of cleft disease in LMIC;
- There is a small body of evidence to suggest that surgical interventions can be done sustainably in LMIC.

### What this study adds

- Cleft disease can be surgically repaired for a low cost in eastern and central Africa;
- Dedicated programs that provide essential CLP surgery can produce substantial clinical and economic benefits;
- Well structured surgical programs can be as cost effective as commonly funded global health interventions.

## Competing interests

The authors declare no competing interests.
